# Identification of dietary patterns by factor analysis and study of the relationship with nutritional status of rural adolescents using factor scores

**DOI:** 10.1186/s41043-015-0009-x

**Published:** 2015-05-01

**Authors:** Kodavalla Venkaiah, Ginnela Narsimhachary Veera Brahmam, Kamasamudram Vijayaraghavan

**Affiliations:** 1 Division of Biostatistics, National Institute of Nutrition, Indian Council of Medical Research (ICMR), Jamai-Osmania, PO, Hyderabad, 500007 India; 2 Division of Community Studies, National Institute of Nutrition, Indian Council of Medical Research (ICMR), Jamai-Osmania, PO, Hyderabad, 500007 India; 3 Department of Biostatistics, National Institute of Nutrition (ICMR), Jamai-Osmania, PO, Hyderabad, 500007 Andhra Pradesh India

**Keywords:** Dietary patterns, Adolescents, Nutritional status, Factor analysis

## Abstract

Study was undertaken to know food and nutrient consumption patterns and their relationship with nutritional status among rural adolescents in Orissa. It was a Community based cross sectional study, conducted at district level in the State of Orissa. Data on 686 adolescent boys and 689 adolescent girls were utilized. Factor analysis was used to find dietary pattern and discriminate analysis and its relationship with undernutrition. The study revealed that among adolescent boys, there existed six patterns among food-groups and three patterns among nutrients explaining 52% and 76% of total variation. Similarly among adolescent girls, seven patterns among food groups and three patterns among nutrients, explaining 67% and 80% of total variation. The discriminate analysis using the factor scores revealed overall 56% of adolescent boys, and 53% of girls were correctly classified. About 46% of boys who were actually thin were predicted as normal, while, 40% who were normal were predicted as thin. Among girls 50% who were actually thin were predicted as normal, while, 36% who were normal were predicted as thin. In conclusions, there exists considerable relationship between dietary patterns and nutritional status among rural adolescents.

## Background

Adolescence is a period of rapid growth and human development, after infancy [1]. Adequate intake of foods and nutrients contribute significantly to the growth and development during the adolescence period particularly among the girls, the future mothers [2]. The complexity of relationship of dietary patterns presents a challenge to study the prevalence of under nutrition in relation to dietary intakes. Food intake is generally studied in terms of adequacy of nutrients [3]. However, foods contain other chemical compounds, some of which are established, some are poorly characterized, and others being completely unknown cannot be measured. Since there is very little information about dietary intakes and the relationship with nutritional status of adolescent population in India [4], an attempt was made to find the relationship between dietary intakes and nutritional status.

About 45% of rural adolescents in India are currently reported to suffer from undernutrition, as assessed by Body Mass Index (BMI) < −2SD [5]. The relationship between intake of a food-group and the prevalence of under nutrition may erroneously be attributed to a single component, overlooking the fact that there exists multi-colinearity between nutrients and foods, which can be demonstrated by employing sophisticated statistical procedure, such as principal components and factor analysis, to derive food consumption patterns [6]. Factor analysis is used to find latent variables or factors among observed variables [7]. In other words, factor analysis is used to reduce the number of variables which groups variables with similar characteristics together. The reduced number of factors can also be used for further analysis. Thus, using Factor analysis it is possible to study the food and nutrient intake patterns and its relationship with the nutritional status. For developing general descriptions of dietary patterns, Principal Component Analysis (PCA), followed by factor analysis is used. The objective is to transform a large set of correlated variables into smaller sets of non-correlated variables, known as principal components or factors [8]. In factor analysis, rather than establishing a diet indicator, data objectively indicate as to how measurements are clustered. The aim of this technique is to identify the underlying structure in data matrix, by summarizing and consigning data to arrive at a systematic measurement of the diet. To summarize data, factor analysis desires dimension that, when interpreted and understood, describes data in terms of a much smaller number of items than do the individual variables [8].

The objective of the present analysis is to describe the food and nutrient-consumption patterns among adolescent rural population that are apparently homogeneous, and to relate these with the prevalence of thinness in the State of Orissa, India using factor analysis.

## Methods

### Study design and sample

The data on diet and nutritional status, collected from the survey of district nutrition profile in Orissa state,was used. In total, 12,000 households— 400 households per district— from all the 30 districts in Orissa state were covered [9]. Anthropometric measurements, viz. height and weight, were taken on all the available individuals in the households. In every alternate household covered for nutrition assessment, a 24-hour recall family diet survey was carried out. Individual dietary intakes were also assessed on 12,621 individuals of different ages of both the sexes. From the average daily intake of foods, nutrients were computed using food composition tables [10]. The present analysis included data on 686 adolescent boys and 689 adolescent girls, aged between ≥ 10 and < 18 years, on whom the data on both anthropometry and dietary intakes was available.

### Data collection

Data was collected by qualified staffs (nutritionist, social worker and anthropologist) that were trained for a period of 3 weeks in standard survey methodologies. Anthropometric measurements such as height and weight were collected using standard equipment and procedures [11]. Height was measured using anthropometer rod and weight was measured using SECA weighing balance. Diet survey was carried out using 24 hr recall method of diet survey in alternative house hold covered for anthropometry [12]. Scientist from the Institute had revisited the household completed on previous day by the project staff to ensure quality control.

### Statistical analysis

The mean, median, and inter-quartile range of various food-groups and nutrients for adolescent boys and girls were calculated. Diet patterns were obtained by exploratory factor analysis for 13 food-groups and 11 (3 macro and 8 micro) nutrients. Further, the proportion of adolescents consuming < 50% of Recommended Dietary Intake (RDI) [13] for foods and nutrients was calculated for different age groups of both the sexes.

Exploratory Factor analysis—an explorative multivariate statistical technique—was used for the identification of factors in a set of dietary measurements. Such factors would correspond to indicators, and all variables were considered simultaneously, each one in relation to the others. Principal Component Analysis was used for extraction of factors and orthogonal rotation (varimax option) to derive non-correlated factors and minimize the number of indicators that have high loading on one factor [6, 7]. The first component extracted is the one that accounts for the maximum possible variance in the dataset. The second component, independent of the first, will be the one that explains the largest possible share of the remaining variance and so on, without the components being correlated with each other [8].

Since dietary data has been collected using 24 hr recall method actual intakes were measured. When quantitative data is available, factor analysis is the suitable statistical method and gives better results as compared to Food Consumption Score, Dietary Diversity score where in these methods uses scoring procedure instead of actual diet intakes. Since we did not aim to compare various methods to determine dietary intake patterns, the other methods have not been applied.

The adequacy of data was evaluated based on the value of Kaiser-Meyer-Olkin (KMO) and Bartlett's test (homogeneity of variance). The KMO measure, which represents the adequacy of sample-size, compares the value of partial correlation coefficients against the total correlation coefficients.

Undernutrition of a given individual was computed on the basis BMI of a given individual (weight in kg/height in metre^2^). The adolescents were categorized into one of the two groups of ‘Thinness’ as Group 1: BMI < median −2 SD units and Normal if BMI ≥ median -2SD units as Group 2 using WHO reference values [14]^.^

Discriminate function analysis was used for studying the relationship between the food and nutrient intake and the nutritional status [8]. The factor scores obtained by the factor analysis for food and nutrients were considered as continuous independent variables, and BMI categories of the adolescents were considered dichotomous dependent variable. Statistical analysis was performed using the SPSS software (version 19.0).

### Ethical approval

The study was approved by the Institutional Ethical Review Committee and Scientific Advisory Committee (SAC) of the National Institute of Nutrition. The study does not involve any biochemical investigations; hence only informed written consent was obtained from the head of the village and from head of the household.

## Results

The average and interquartile range of various food intakes according to sex is presented in Table 1. The food grouping was done as is adopted in the National Nutrition Monitoring Bureau surveys in India over the past 40 years and is well-accepted by nutritionists. The mean and median intakes of were comparable in both the boys and girls with respect to cereals & millets, pulses and legumes, roots and tubers, other vegetables, condiments and spices, and fats and oils . However, the median intakes of nuts and oil seeds, fruits, fish and other sea foods, meat and poultry, milk and milk products and sugar and *jaggery* indicated that 50% were consuming these foods inadequately. In fact, 75% of the adolescents were consuming income elastic foods like fish and other seafood’s, meat and poultry and milk and milk products in amounts < 50% of Recommended dietary intakes(RDI). However the intakes are more or less similar in both adolescent boys and girls.

**Table 1:** **Average intake of foods among rural adolescent population by sex**

**Food-group (g/day)**	**Sex**	**Mean**	**SD**	**Percentiles**		
				**25**	**50**	**75**
Cereals and millets	Boys	456.7	195.6	315.8	445.5	584.0
	Girls	449.4	180.3	325.4	437.9	559.7
Pulses and legumes	Boys	26.9	37.2	0.0	17.8	38.4
	Girls	28.2	36.8	0.0	20.7	41.4
Green leafy vegetables	Boys	27.0	50.8	0.0	0.0	38.6
	Girls	29.6	55.2	0.0	0.0	47.5
Roots and tubers	Boys	77.5	68.3	26.9	62.6	111.2
	Girls	72.3	65.4	20.0	61.1	103.5
Other vegetables	Boys	70.5	71.8	0.0	55.3	106.5
	Girls	62.1	67.1	0.0	47.9	91.4
Nuts and oilseeds	Boys	0.91	5.6	0.0	0.0	0.3
	Girls	1.1	7.8	0.0	0.0	0.3
Condiments and spices	Boys	5.9	10.1	1.9	3.6	6.6
	Girls	5.5	7.9	1.9	3.8	6.5
Fruits	Boys	13.1	27.5	0.0	0.0	14.7
	Girls	11.2	24.9	0.0	0.0	11.0
Fish and other sea-foods	Boys	9.8	27.9	0.0	0.0	0.0
	Girls	10.1	30.0	0.0	0.0	0.0
Meat and poultry	Boys	5.0	31.2	0.0	0.0	0.0
	Girls	1.9	12.6	0.0	0.0	0.0
Milk and milk products	Boys	13.8	42.4	0.0	0.0	0.0
	Girls	12.8	37.1	0.0	0.0	0.0
Fats and oils	Boys	6.6	7.2	4.6	2.5	8.2
	Girls	6.4	11.3	4.4	2.5	7.7
Sugar and *jaggery*	Boys	11.1	22.2	0.0	0.0	14.7
	Girls	10.3	19.7	0.0	0.0	12.9

n:Boys-686 and Girls-689.

The median intakes of various nutrients according to age groups and sex are presented in Table 2. The median intake of energy for boys and girls were 1902 Kcal and 1844 Kcal respectively. There exists a large variation in the consumption of various nutrients was observed among adolescents. There were no sex differentials in the median intakes of various nutrients.

**Table 2:** **Average intake of nutrients among rural adolescent population by sex**

**Nutrients**	**Sex**	**Mean**	**SD**	**Percentiles**		
				**25**	**50**	**75**
Protein (g)	Boys	44.7	20.6	30.5	42.4	55.6
	Girls	43.9	18.4	31.5	41.6	52.7
Total fat (g)	Boys	11.6	10.0	5.7	8.9	13.9
	Girls	11.3	13.0	5.8	8.5	13.3
Energy (Kcal)	Boys	1929	773	1383	1902	2414
	Girls	1895	695	1426	1844	2279
Calcium (mg)	Boys	341.0	440	126.4	224	399.4
	Girls	337.0	680	121.8	222	393.6
Iron (mg)	Boys	11.6	8.9	6.7	9.4	13.5
	Girls	11.4	8.1	6.7	9.3	13.4
Vitamin A (mg)	Boys	276	553	26.4	54.6	171.1
	Girls	302	634	23.5	52.7	196.5
Thiamine (mg)	Boys	1.2	0.5	0.8	1.2	1.6
	Girls	1.2	0.5	0.9	1.2	1.6
Riboflavin (mg)	Boys	0.4	0.2	0.3	0.4	0.6
	Girls	0.4	0.2	0.3	0.4	0.5
Niacin (mg)	Boys	19.0	8.1	12.8	18.4	24.6
	Girls	18.8	7.6	13.4	18.1	23.3
Vitamin C (mg)	Boys	58.8	71.1	18.1	34.9	69.8
	Girls	58.6	82.2	16.7	32.5	72.5
Free Folic acid (μg)	Boys	122	81.1	68.2	104.7	153.5
	Girls	122	76.2	72.5	109.2	148.2

n:Boys-686 and Girls-689.

Since the Recommended dietary intakes (RDIs) are different for different age groups and sex, the intakes were compared with RDI according to age groups and sex (Table 3). About 7 to 16% of adolescents were not meeting even 50% of RDI for cereals. In case of pulses more than a half of the adolescents were not meeting 50% RDI. Improving trend with age in the consumption of different foods was observed in both the boys and girls, with no significant sex differentials.

**Table 3:** **Percent (%)of adolescent consuming foods in amount <50% of RDI**

**Age group (y)**	**Sex**	**n**	**Cereals**	**Pulses**	**Leafy-veg.**	**Other-veg.**	**Milk and milk products**	**Fats and oils**	**Sugar and Jaggery**
10-12	Boys	310	16.1	58.7	82.6	36.1	97.1	85.8	71.9
	Girls	278	14.0	50.7	75..2	36.3	95.7	84.2	74.8
13-15	Boys	246	10.6	61.4	80.1	26.4	95.1	80.5	67.5
	Girls	262	6.5	55.3	79.4	28.2	94.7	88.9	66.0
16-17	Boys	130	7.7	46.9	75.4	23.8	94.6	78.5	59.2
	Girls	149	8.1	47.7	78.5	32.9	95.3	85.2	67.8
All Boys		686	12.5	57.4	80.3	30.3	85.9	82.5	67.9
All Girls		689	9.9	51.8	77.5	32.5	95.2	86.2	70.0
Boys + Girls pooled		1375	11.2	54.6	78.9	31.4	95.6	84.4	68.9

The consumption of energy was relatively better with 86% consuming > 50% of RDA, while about 70% were consuming proteins > 50% of RDA (Table 4). More than three fourths of the adolescent boys and girls consumed less than 50% of the RDA for fats and micronutrients such as calcium, riboflavin iron and vitamin A.

**Table 4:** **Percent of adolescents consuming nutrients < 50% of RDA**

**Age group**	**Gen-der**	**n**	**Protein**	**Energy**	**Calcium**	**Iron**	**Vit-A**	**Thia- min**	**Ribo-flavin**	**Niacin**	**Vit-C**	**Folic acid**	**Fat**
10-12	Boys	310	39.7	20.6	84.5	82.9	84.2	10.6	89.4	9.4	59.7	37.4	93.2
	Girls	115	31.4	16.9	82.0	84.2	79.5	10.8	89.2	9.0	57.6	31.3	92.1
13-15	Boys	246	28.5	13.0	79.3	84.1	83.3	7.3	84.1	4.9	53.3	30.9	89.8
	Girls	262	27.1	8.8	83.2	76.3	81.7	6.5	83.2	5.0	58.8	24.4	91.2
16-17	Boys	130	14.6	8.5	78.5	73.1	81.5	5.4	70.8	3.8	46.9	14.6	86.2
	Girls	149	21.5	7.4	83.2	80.5	83.2	4.7	77.9	2.0	57.7	24.2	90.6
All Boys		686	30.9	15.6	81.5	81.5	83.4	8.5	84.0	6.7	55.0	30.8	90.7
All Girls		689	31.6	11.8	82.7	80.4	81.1	7.8	84.5	6.0	58.1	27.1	91.4
Boys + Girls pooled		1375	31.3	13.7	82.1	80.9	82.3	8.1	84.2	6.3	56.5	28.9	91.1

There was sufficient correlation between different foods (Boys: 0.613; Girls: 0.513) to proceed with factor analysis. Similarly, in the case of nutrients, the KMO measure indicated a higher correlation between nutrients (boys: 0.793 and girls: 0.755). The Bartlett test of sphericity for foods and nutrients among boys and girls was highly significant (p < 0.01), indicating homogeneity of variance by the consumption of foods and nutrients.

Five components for boys and seven components for girls were extracted by factor analysis for different food-groups (Table 5). The five components (factors) in the initial solution have an Eigen value over 1, accounting for about 52% of the observed variation in the food-consumption pattern among the boys, while, among the girls the seven factors accounted for about 67% of the observed variation in the food-consumption pattern.

**Table 5:** **Rotated component matrix for food-groups**

**Food-group (g)**	**Adolescent boys component**					**Adolescent girls component**						
	**1**	**2**	**3**	**4**	**5**	**1**	**2**	**3**	**4**	**5**	**6**	**7**
Cereals and millets	0.00	0.55	0.13	0.31	0.07	−0.18	−0.04	0.23	0.68	0.28	−0.01	0.03
Pulses andlegumes	0.46	0.17	−0.19	0.20	−0.16	0.11	0.00	0.00	0.07	0.84	0.00	−0.05
Green-leafy vegetables	−0.02	0.70	−0.14	−0.17	−0.11	−0.00	−0.08	0.04	0.26	−0.28	−0.21	−0.58
Roots and tubers	0.16	−0.12	0.12	0.62	−0.16	0.05	0.20	0.75	−0.03	−0.10	0.07	−0.04
Other vegetables	0.10	0.07	−0.16	0.73	0.18	0.07	−0.11	0.76	0.07	0.10	−0.07	0.11
Nuts and oilseeds	0.02	0.03	−0.00	0.02	0.89	−0.00	−0.03	0.11	0.16	−0.22	−0.10	0.79
Condiments and spices	−0.24	0.17	0.56	0.24	0.13	−0.06	−0.00	0.15	0.35	−0.24	0.54	−0.18
Fruits	0.25	0.62	0.11	−0.04	0.09	0.19	0.10	−0.14	0.72	−0.09	0.06	−0.01
Fish and other sea-foods	0.14	−0.20	0.63	−0.20	0.16	−0.01	0.83	−0.12	0.12	−0.12	−0.08	0.08
Meat and poultry	0.05	0.09	0.57	−0.00	−0.23	0.06	0.03	−0.07	−0.06	0.11	0.80	0.11
Milk and milk products	0.64	−0.08	0.10	0.11	0.01	0.81	−0.02	0.10	−0.07	−0.01	0.15	−0.02
Fats and oils	0.59	0.15	0.35	0.31	−0.08	0.09	0.77	0.26	−0.06	0.16	0.14	−0.06
Sugar and jaggery	0.75	0.12	−0.08	−0.05	0.14	0.79	0.09	0.02	0.11	0.14	−0.12	0.03
Eigen value	2.16	1.31	1.23	1.09	1.02	1.58	1.35	1.26	1.19	1.05	1.0	1.0
Variance explained	16.6	10.1	9.5	8.4	7.9	13.7	10.4	9.7	9.2	8.0	7.7	7.7

Extraction method; Principal component analysis; Rotation method; Varimax with Kaiser normalization; Rotation converged in 5 iterations among Boys and in 7 iterations among Girls.

The first factor, which accounted for 16.6% of the total variance among the boys, was labeled as *income-elastic foods*, as high factor loading was observed for sugar and *jaggery*, followed by milk and milk products, Fats and oils and pulses and legumes. The second factor explained 10.1% of the total variance and was labeled as *plant-foods*, and green leafy vegetables, fruits and cereals and millets were the major contributors. The third factor accounted for 9.5% of the total variance, and this factor was labeled as *traditional* contributed by the intake of fish and other sea foods, meat and poultry and condiments and spices. The fourth factor explained 8.4% of the total variance and labeled as *plant foods* contributed by other vegetables and roots and tubers. The last and the fifth factor explained 7.9% of the total variance and were labeled as *protein-rich foods* (nuts and oilseeds).

In case of the girls, the first factor, which accounted for 13.7% of the total variance, was labeled as *income-elastic foods* with high factor loadings for milk and milk products followed by sugar and *jaggery*. The second factor explained 10.4% of the total variance and was labeled as *traditional* (Fish and other sea-foods and Fats and oils). The third factor accounted for 9.7% of the total variance, and was labeled as plant foods characterized by the intake of other vegetables and roots and tubers. The fourth factor explained 9.2% of the total variance and labeled as *staple* such as cereals and millets along with fruits. The fifth factor explained 8.0% of the total variance and were labeled as *plant-protein* (pulses and legumes).The sixth and seventh factors explains 7.7% of total variation each consists of meat and poultry, condiments and spices, nuts and oil seeds and green leafy vegetables (Table 5).

In the case of nutrients, three components for each sex were extracted (Table 6). Energy, thiamin, niacin, protein, riboflavin, and free folic acid had higher loadings on factor 1 among the boys, which explained 51.8% of the total variance. It was labeled as *macro nutrients and B-vitamins*. Factor 2, labeled as ‘*vitamins*’ contained vitamin A and C and had high loadings explaining 14.5% of the total variation. The third factor characterized by the intake of calcium and iron, labeled as ‘*minerals’* which accounted for 9.3% of the total variance. In case of girls, factor 1 explained 47.2% of total variance with loadings of similar to that observed in boys. The second factor which explained 17% of total variation was labeled as *fats and minerals* (Calcium and total fat) and the third factor explaining 15.4% of total variation was labeled as *macro nutrients and vitamins*.

**Table 6:** **Rotated component matrix for nutrients**

**Nutrient**	**Adolescent boys component**			**Adolescent girls component**		
	**1**	**2**	**3**	**1**	**2**	**3**
Proteins (g)	0.86	0.04	0.35	0.82	0.45	0.05
Total fat (g)	0.48	0.18	0.34	0.17	0.91	−0.00
Energy (Kcal)	0.93	0.05	0.13	0.92	0.21	−0.01
Calcium (mg)	0.02	0.16	0.87	0.00	0.92	0.16
Iron (mg)	0.39	0.00	0.60	0.38	0.45	0.05
Vitamin A (mg)	0.10	0.90	0.16	0.00	0.07	0.93
Thiamine (mg)	0.93	0.07	0.13	0.95	0.05	0.06
Riboflavin (mg)	0.77	0.41	0.21	0.71	0.14	0.50
Niacin (mg)	0.92	0.06	0.04	0.92	−0.00	0.03
Vitamin C (mg)	0.15	0.87	0.04	0.07	0.03	0.87
Free folic acid (μg)	0.71	0.35	0.07	0.61	0.14	0.51
Eigen value	5.7	1.6	1.0	5.2	1.8	1.8
Variance explained	51.8	14.5	9.3	47.2	17.0	15.4

Extraction method; Principal component analysis; Rotation method; Varimax with Kaiser normalization; Rotation converged in 3 iterations in both Boys and Girls.

The prevalence of thinness (BMI < −2SD) was 49.4% among the boys and 46.6% among the girls.

The distributions of predicted thinness based on the derived BMI values by the discriminate function analysis using the factor scores of various foods and nutrients as against those based on the anthropometric measurements are presented in Table 7. The discriminate analysis revealed overall 56% of adolescent boys, and 53% of girls were correctly classified. About 46% of boys who were actually thin were predicted as normal, while, 40% who were normal were predicted as thin. Among girls 50% who were actually thin were predicted as normal, while, 36% who were normal were predicted as thin.

**Table 7:** **Classification of subjects according nutritional status with undernutrition and without undernutrition using observed BMI and predicted BMI using scores derived from factor analysis for foods and nutrients**

**a. Classification results: adolescent boys**					
BMI			Predicted BMI group according to factor scores derived through factor analysis		Total
			<−2SD	≥ − 2SD	
According to BMI	n%	<−2SD	250	216	466
		≥ − 2SD	89	131	220
		<−2SD	53.6	46.4	100
		≥ − 2SD	40.5	59.5	100
**Factor scores derived by factor analysis predicted correctly (55.9%) those who are thin as thin and normal as normal group. Adolescent boys (46.4%), who were originally thin (<−2SD) were predicted as normal and 40.5% who were originally normal were predicted as thin.**					
**b. Classification results: adolescent boys**					
BMI			Predicted BMI group according to factor scores derived through factor analysis		Total
			<−2SD	≥ − 2SD	
According to BMI	n%	<−2SD	264	268	532
		≥ − 2SD	57	100	157
		<−2SD	49.6	50.4	100
		≥ − 2SD	36.6	63.7	100

Factor scores derived by factor analysis predicted correctly (52.8%) those who are thin as thin and normal as normal group. Adolescent girls (50.4%), who were originally thin (<−2SD) were predicted as normal and 36.3% who were originally normal were predicted as thin.

## Discussion

Studies have shown that conventional dietary pattern has some limitations to study the relationship between dietary patterns and type 2 diabetes as well as cardiovascular diseases [15, 16]. Our earlier study has established relationship between factor scores of dietary patterns derived through factor analysis and chronic energy deficiency [17]. In the present manuscript similar technique has been used to demonstrate the relationship between dietary pattern and nutritional status among adolescents.

The dietary data has been collected using 24 hr recall method in which actual intakes were measured. When quantitative data is available, the multivariate factor analysis which is based on interrelations of the entire data set is a suitable statistical method. The result obtained through this method is robust in indentifying the dietary patterns.

It is interesting to note that a half of the rural adolescents were not consuming green leafy vegetables, nuts and oil seeds, fruits, fish and other fish products, meat and poultry, milk and milk products and sugar and *jaggery*. Low consumption of green leafy vegetables among adolescents may be due to non availability and low purchasing capacity among the study population. Consequently, about 70-90% of them were not meeting even 50% of RDI for various nutrients such as calcium, iron, vitamin A and riboflavin [18]. Results of an earlier study revealed that both PCA and cluster analysis are useful approaches for the assessment of dietary patterns [19]. A common criticism of the two techniques is that these involve several subjective —but important— decisions, such as grouping of foods and nutrients and possible transformations of variables [20]. PCA also involves decisions about the number of components to be retained and their subsequent labeling [20]. Another disadvantage of these techniques is that though they generate patterns based on variation in diet, there is no guarantee that these patterns will be predictive of a particular health outcome. However, the techniques have the advantage that they are empirically derived and are, therefore, not limited by mere *a priori* knowledge [8].

In developing countries, studies on identifying dietary patterns and their relationship with nutritional status are scarce. The intake of foods and nutrients highly correlated, and the classification methods based on univariate analysis may lead to flawed estimates [8]. Therefore, multivariate methods, such as factor analysis, represent an alternative approach to the evaluation of individual foods and nutrients and to examine the association of dietary patterns with nutritional status. Moreover, it should be kept in mind that individuals intake of nutrients depend on their intakes of different types foods, which are influenced by many factors, such as cultural, socioeconomic and demographic characteristics. Describing food intake in different consumption patterns may be useful in developing community based intervention programmes. It is possible to find a smaller number of measures using factor analysis that would permit the identification of persons who are nutritionally at risk [21].

Several studies examined the relationship between dietary patterns and nutritional status, overweight, obesity etc., there were many in consistencies in establishing a clear relationship between them [22, 23]. However, the present data showed a considerable relationship between the dietary pattern and the nutritional status, as indicated by the distribution of predicted BMI based on factor scores. About a half of the adolescents of with thinness (boys: 54% and girls: 50%) were correctly classified based on the scores obtained by factor analysis. Similar findings were also observed among adult population from our earlier study [17].

National Institute of Nutrition conducted workshop to disseminate the results of the survey to all the stakeholders of Government of Orissa and distributed brochures containing the need for consumption of locally available foods such as green leafy vegetables and fruits. This will be useful for the politician, policy planners or development partners in formulating optimal dietary interventions to this vulnerable population [24].

## Conclusion

Factor analysis allowed the identification of dietary patterns based on the data from food and nutrient intake. The factors extracted for boys and girls are different in case of food groups, but similar in case of nutrients among adolescent rural population.

There exists a relationship between specific diet pattern and thinness among the rural adolescent population. These results will be useful for identifying adolescent boys and girls with thinness in the community and help the planners in formulating dietary interventions to them.
